# Nutrition and Exercise Interventions During Hospitalization in Frail or Sarcopenic Patients: A Scoping Review of Intervention Configurations and Evidence Gaps

**DOI:** 10.3390/nu18121994

**Published:** 2026-06-19

**Authors:** Shinichi Watanabe, Takayasu Koike, Kenji Tsujimoto, Ryoma Tahara, Tomohiko Kamo, Katsuyoshi Suzuki, Keisuke Suzuki

**Affiliations:** 1 Department of Physical Therapy, Faculty of Rehabilitation, Gifu University of Health Science, Gifu 500-8281, Japan; billabonghonor@yahoo.co.jp (S.W.); takayasukoike82@gmail.com (T.K.); tsujiken1260@gmail.com (K.T.); 2 Department of Rehabilitation, National Hospital Organization Nagoya Medical Center, Nagoya 460-0001, Japan; 3 Department of Occupational Therapy, Faculty of Rehabilitation, Gifu University of Health Science, Gifu 500-8281, Japan; r-tahara@gifuhoken.ac.jp; 4 Department of Physical Therapy, Faculty of Rehabilitation, Gunma Paz University, Takasaki 370-0006, Japan; kamo@paz.ac.jp; 5 Department of Rehabilitation, Shizuoka Cancer Center, 1007 Shimonagakubo, Nagaizumi-cho, Shizuoka 411-8777, Japan; katsu.suzuki@scchr.jp

**Keywords:** frailty, sarcopenia, hospitalization, nutrition, exercise, rehabilitation, physical function

## Abstract

**Background/Objectives**: Frailty and sarcopenia are common among hospitalized patients and are associated with poor clinical outcomes. Nutritional and exercise interventions are widely used to prevent muscle loss and functional decline; however, their independent and incremental effects remain unclear. This scoping review aimed to systematically map the characteristics and reported effects of these interventions during hospitalization. **Methods**: This scoping review followed the Joanna Briggs Institute methodology and the PRISMA-ScR guidelines. A comprehensive literature search was conducted in PubMed/MEDLINE, EMBASE, CENTRAL, and PEDro. Eligible studies included adult hospitalized patients receiving nutritional interventions, exercise interventions, or both. Interventions were categorized into four groups: no intervention, nutrition alone, exercise alone, and combined interventions. Data regarding study characteristics, intervention details, and clinical outcomes were extracted and descriptively summarized. **Results**: Thirty-three studies were included. Considerable heterogeneity was observed in patient populations, intervention characteristics, and outcome measures. Most studies evaluated configurations including an exercise component (exercise alone or combined nutrition–exercise), whereas studies isolating nutrition or providing direct head-to-head comparisons between combined and single-component configurations were limited. Intervention dose and reporting were highly variable across studies. **Conclusions**: Current evidence on the effects of nutritional and exercise interventions during hospitalization remains heterogeneous and limited. Future studies should adopt standardized intervention reporting and directly compare combined and single-component strategies to determine additive and synergistic effects in patients with frailty or sarcopenia.

## 1. Introduction

Frailty and sarcopenia are highly prevalent among hospitalized patients and are strongly associated with adverse clinical outcomes, including functional decline, prolonged length of hospital stay, institutionalization, and increased mortality [1,2,3]. These conditions frequently worsen during hospitalization due to a combination of physical inactivity, systemic inflammation, bed rest, and insufficient nutritional intake, particularly in acutely ill and critically ill populations [4,5]. Hospitalization itself is recognized as a major precipitating factor for rapid muscle loss and functional deterioration [6]. Therefore, interventions targeting both muscle preservation and functional recovery are essential, and nutritional therapy together with exercise-based rehabilitation are widely considered key components of comprehensive care [3,7].

Nutritional interventions, especially those ensuring adequate protein and energy intake, play a central role in maintaining muscle protein synthesis and attenuating catabolic processes [8]. In parallel, exercise interventions—including resistance training, mobilization, and structured rehabilitation programs—are well established to improve muscle strength, physical performance, and activities of daily living [9,10]. Previous studies and systematic reviews have suggested that combining nutritional support with exercise may lead to greater improvements in muscle mass and function compared with either intervention alone [11,12]. However, the current body of evidence remains limited and highly heterogeneous, with substantial variation in patient characteristics, timing of intervention initiation, nutritional composition, exercise dose, and outcome measures [12,13].

Importantly, most existing studies evaluate combined nutrition and exercise interventions as a single package, without clearly distinguishing the independent effects of each component [12]. As a result, it remains unclear whether the observed benefits are primarily attributable to exercise, nutritional support, or their interaction. Furthermore, few studies directly compare combined interventions with single-component strategies, making it difficult to determine whether combined approaches provide additional or incremental benefits beyond either nutrition or exercise alone [13]. This lack of differentiation limits the ability to establish optimal intervention strategies, hinders clinical decision-making, and restricts efficient allocation of healthcare resources in hospital settings.

To address this gap, it is necessary to adopt a conceptual framework that categorizes interventions into four groups: no intervention, nutrition alone, exercise alone, and combined nutrition and exercise. Such a framework enables systematic mapping of which intervention configurations have been studied and which comparisons remain underrepresented in the literature. Given that the available studies address fundamentally different comparisons rather than a single common contrast, and report non-commensurable outcomes across diverse clinical populations, a scoping review is the most appropriate methodology to map the structure of this evidence. Therefore, the aim of this review is to systematically map the characteristics, implementation, and comparison structures of nutritional and exercise interventions during hospitalization in patients with frailty or sarcopenia, and to identify gaps in the available evidence specifically, which intervention configurations have been compared directly and which remain insufficiently studied. Consistent with scoping review methodology, the aim is to characterise the evidence landscape rather than to estimate the relative effectiveness of interventions, which would require systematic review with meta-analysis.

## 2. Materials and Methods

### 2.1. Study Design

This scoping review was conducted in accordance with the methodological framework proposed by the Joanna Briggs Institute for scoping reviews and is reported following the PRISMA Extension for Scoping Reviews (PRISMA-ScR) [14]. The aim of this review was to systematically map the characteristics and reported effects of nutritional and exercise interventions during hospitalization in patients with frailty or sarcopenia, with a particular focus on mapping the comparison structures available in the literature and identifying evidence gaps. The protocol for this scoping review was prospectively registered in the Open Science Framework (OSF Registries) (registration date: 11 April 2026; https://osf.io/58v7p/, accessed on 11 April 2026).

### 2.2. Participants

Studies were eligible if they included adult patients (aged ≥ 18 years) who were hospitalized in acute care units, rehabilitation wards, or intensive care units. Studies were included regardless of whether frailty or sarcopenia was formally diagnosed, provided that the population was described as frail, sarcopenic, or at risk of these conditions (e.g., muscle weakness, functional decline, or malnutrition).

### 2.3. Concept

This review included studies that examined nutritional interventions, exercise interventions, or their combination during hospitalization. Nutritional interventions encompassed protein supplementation, oral nutritional supplements, enteral or parenteral nutrition, and dietary management strategies. Exercise interventions included resistance training, mobilization, structured rehabilitation programs, and physical therapy. To map the comparison structures available in the literature, interventions were conceptually categorized into four groups: Group A (no nutrition and no exercise), Group B (nutrition alone), Group C (exercise alone), and Group D (combined nutrition and exercise). Where possible, studies were further examined in terms of comparisons between these groups, such as A versus D, B versus D, and C versus D, to characterise which intervention configurations have been directly compared in the literature.

### 2.4. Context

The context was limited to hospital-based settings, including acute care hospitals, rehabilitation facilities, and intensive care units. Studies conducted exclusively in community or outpatient settings were excluded.

### 2.5. Types of Sources

This review included randomized controlled trials, non-randomized interventional studies, observational studies, and pilot or feasibility studies. Conference abstracts without sufficient methodological detail were excluded.

Studies were excluded if they were conducted exclusively in outpatient or community settings; were conference abstracts or study protocols lacking outcome data; enrolled non-adult populations (<18 years); did not include an identifiable nutritional and/or exercise intervention; or were not available in English or Japanese.

### 2.6. Search Strategy

A comprehensive literature search was conducted in the following electronic databases: PubMed/MEDLINE (11 April 2026), EMBASE (10 April 2026), CENTRAL (13 April 2026), and PEDro (15 April 2026). The search strategy combined controlled vocabulary and free-text terms related to hospitalization, frailty or sarcopenia, nutritional interventions, and exercise or rehabilitation.

Each database was searched from inception to the final search date, with no restriction on publication start date.

Prior to protocol registration, a pilot search in PubMed/MEDLINE was performed on 4 April 2026 to refine the search strategy. The protocol was then finalised and registered in OSF on 11 April 2026, after which the protocol-aligned database searches reported above were performed.

The search strategy was developed based on an initial limited search and refined iteratively. Reference lists of included studies and relevant reviews were also screened to identify additional studies. Grey literature, including clinical trial registries, was considered to minimize publication bias. Detailed search strategies for each database are provided in the Supplementary Materials (Supplementary Table S1).

### 2.7. Selection of Sources

All identified records were imported into the systematic review management software Rayyan (Rayyan Systems Inc., Cambridge, MA, USA), and duplicates were removed. Two reviewers (S.W. and K.S. (Keisuke Suzuki)) independently screened titles, abstracts, and full texts for eligibility. Disagreements were resolved through discussion with a third reviewer (T.K. (Takayasu Koike)) when necessary. The study selection process was documented using a PRISMA-ScR flow diagram [14]. The eligibility criteria were developed according to the Population–Concept–Context framework recommended by the Joanna Briggs Institute for scoping reviews.

### 2.8. Data Extraction

Data were extracted using a standardized data extraction form developed specifically for this review. The extracted information included study characteristics (author, year of publication, country, and study design), participant characteristics (age, sex, and clinical condition), and details of the interventions. Nutritional interventions were recorded in terms of type and quantitative parameters such as protein and energy intake, while exercise interventions were characterized by type, frequency, intensity, duration, and timing of initiation. In addition, outcome measures were collected, including muscle mass, muscle strength, physical function, activities of daily living, length of hospital stay, and mortality. Particular attention was given to the quantification of intervention dose, with a focus on both nutritional intake and exercise parameters, to enable a detailed comparison of intervention characteristics across studies.

### 2.9. Data Synthesis

A meta-analysis was not performed because the included studies addressed heterogeneous and non-equivalent comparisons (A vs. B, A vs. C, A vs. D, B vs. D, and C vs. D) and reported outcomes on differing scales across distinct populations. Rather than estimating a single pooled effect lacking a coherent clinical interpretation, our aim was to map these comparison structures and identify evidence gaps; findings were therefore synthesized descriptively, supplemented by structured vote counting (see Section 3.3). Studies were categorized according to the four-group framework (A–D) to enable the evaluation of incremental effects. Results were summarized using tables and graphical representations, including outcome mapping and evidence gap visualization, to illustrate patterns, variability, and gaps in the current literature. To visualize intervention heterogeneity across studies, exercise frequency and intensity were semi-quantitatively categorized into five levels based on the reported intervention characteristics for descriptive mapping purposes. For outcome mapping (Supplementary Figure S1), effect direction was classified according to the original authors’ reported conclusions for the primary outcome of each comparison as positive, neutral (no significant difference), or negative, without re-analysis of effect sizes or confidence intervals.

## 3. Results

### 3.1. Study Selection

**Figure 1:**
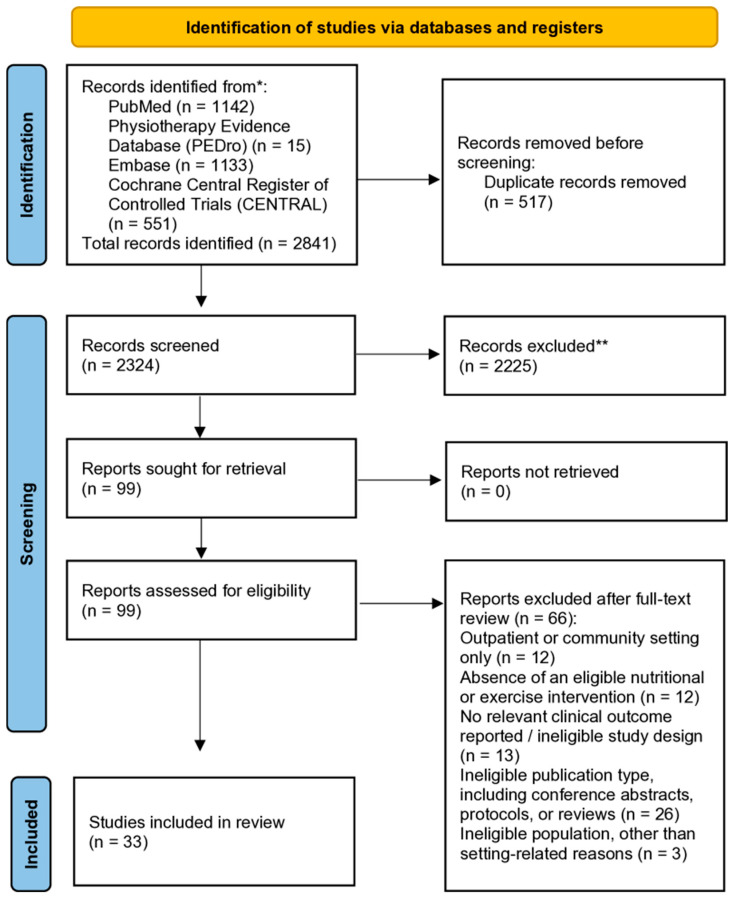
Study selection process for the scoping review.

A total of 2841 records were identified through database searching. After removal of duplicates, 2324 records underwent title and abstract screening. Following full-text assessment of 99 articles, 33 studies were included in this scoping review [15,16,17,18,19,20,21,22,23,24,25,26,27,28,29,30,31,32,33,34,35,36,37,38,39,40,41,42,43,44,45,46]. The study selection process and reasons for exclusion are presented in Figure 1.

Of the 99 full-text articles assessed for eligibility, 66 were excluded for the following reasons: outpatient or community setting only (*n* = 12); absence of an eligible nutritional or exercise intervention (*n* = 12); no relevant clinical outcome reported or ineligible study design (*n* = 13); ineligible publication type, including conference abstracts, protocols, or reviews (*n* = 26); and ineligible population, other than setting-related reasons (*n* = 3). The remaining 33 studies were included. * indicates the records identified through database searching; ** indicates the records excluded during title and abstract screening.

### 3.2. Characteristics of Included Studies

The characteristics of the included studies are summarized in Table 1. The included studies comprised predominantly randomized controlled trials, along with several cohort, retrospective, pilot, and feasibility studies. Study populations included critically ill intensive care unit patients, frail older adults, patients undergoing rehabilitation, stroke patients, patients with chronic obstructive pulmonary disease, and cancer patients. The studies were conducted across multiple countries, including China, Japan, Denmark, Austria, Italy, Australia, and Canada. Sample sizes ranged from 20 to 529 participants. Hospital settings varied considerably and included acute care hospitals, rehabilitation hospitals, medical and surgical intensive care units, postoperative rehabilitation settings, and inpatient chemotherapy programs. Reported outcomes were heterogeneous and included muscle mass, muscle strength, gait performance, physical function, activities of daily living, quality of life, mortality, intensive care unit-acquired weakness, and length of hospital stay. Detailed intervention characteristics are summarized in Supplementary Table S2.

Among the 29 included randomized controlled trials, PEDro scores ranged from 4 to 9 out of 10 (median 6; mean 6.2), indicating overall moderate-to-good methodological quality. Three trials scored 9, three scored 8, six scored 7, six scored 6, eight scored 5, and three scored 4; no trial scored below 4. Using a conventional categorisation, 3 trials (10%) were rated as excellent (9–10/10), 15 (52%) as good (6–8/10), and 11 (38%) as fair (4–5/10). Per-study PEDro scores are presented in Supplementary Table S3.

### 3.3. Classification of Intervention Structures and Incremental Comparisons

**Figure 2:**
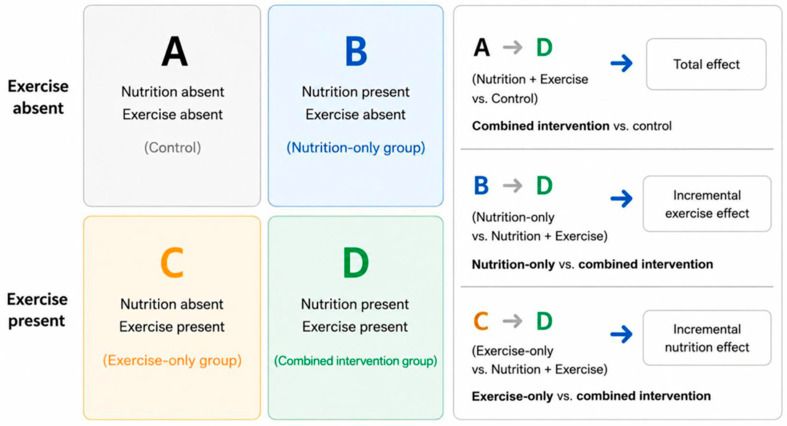
Conceptual Framework for Mapping Nutrition and Exercise Intervention Configurations. The 2 × 2 matrix categorises interventions by the presence or absence of nutrition and exercise components, yielding four groups: Group A (control), Group B (nutrition-only), Group C (exercise-only; shown in orange), and Group D (combined; shown in green). The orange and green highlight the two exercise-containing configurations. The arrows indicate the three comparison structures used to map the evidence: A → D (total effect), B → D (incremental exercise effect), and C → D (incremental nutrition effect).

Studies were classified according to the conceptual framework shown in Figure 2 and categorized into four intervention groups: no intervention (A), nutrition-only intervention (B), exercise-only intervention (C), and combined nutrition and exercise intervention (D). Only a limited number of studies directly compared single-component and combined interventions. Several four-arm randomized controlled trials provided data on more than one comparison structure, including A versus B, A versus C, and A versus D, allowing the distribution of available contrasts to be mapped. Comparisons between exercise-only and combined interventions (C versus D) were more commonly reported than comparisons involving nutrition-only interventions. Some studies demonstrated additional benefits of combined interventions on muscle strength, mobility, physical function, and muscle mass, whereas others reported no clear additive effect beyond exercise alone. Across the included studies, the distribution of evidence was uneven: configurations including an exercise component (Groups C and D) accounted for the majority of intervention arms, while configurations isolating nutrition (Group B) were comparatively rare. Reported outcomes and effect directions varied substantially across studies, populations, and intervention configurations, and these patterns are summarised descriptively rather than as effectiveness estimates. Detailed comparison structures and outcome directions are provided in Supplementary Table S3. Outcome mapping across intervention categories is illustrated in Supplementary Figure S1.

To clarify the structure of comparisons, the included studies were grouped according to the type of direct contrast provided. Fifteen studies compared the combined intervention against no nutritional or exercise intervention (A vs. D); two studies isolated the incremental effect of adding exercise to nutrition (B vs. D); and seventeen studies compared exercise alone with the combined intervention (C vs. D). Because several four-arm and three-arm trials contributed to more than one contrast, these counts are not mutually exclusive.

Among the 17 studies permitting an exercise-versus-combined (C vs. D) comparison, 11 demonstrated an additional benefit of the combined intervention (most commonly on muscle strength, muscle mass, gait speed, or activities of daily living), 1 showed partial benefit confined to a clinical subgroup (SOFA < 10), and 5 showed no additional benefit beyond exercise alone. The most frequently assessed outcomes across studies were muscle strength, activities of daily living (Barthel Index), and physical performance or mobility.

The 11 C versus D contrasts reporting an additional benefit of the combined intervention were conducted predominantly in rehabilitation-phase or post-acute populations, comprising inpatient or post-acute rehabilitation [21,22,24,30,38,41], rehabilitation following hip fracture [17] or chronic respiratory disease [18], cancer-related inpatient care during chemotherapy [23], post-liver-transplantation rehabilitation [28], and a mixed rehabilitation/community setting [16]. Resistance training was incorporated as the principal or partial exercise component in seven of these eleven studies [16,17,22,23,28,30,38], and explicit protein targets of ≥1.2 g/kg/day or ≥20 g/day of whey/protein supplementation were reported in five [21,22,23,28,30] (Supplementary Table S2). Reported favourable effects spanned muscle strength, muscle mass, gait speed, activities of daily living, and—in selected populations—exercise tolerance and quality of life.

The single partial-benefit study (Nakamura et al. 2020 [33]) was conducted in critically ill ICU patients receiving HMB–arginine–glutamine supplementation together with electrical muscle stimulation and rehabilitation initiated on day 2, and reported reduced femoral muscle loss with the combined intervention only in a less severely ill subgroup (SOFA < 10), indicating that the response to nutritional supplementation combined with rehabilitation may be conditional on illness severity in the critically ill population.

The five C versus D contrasts reporting no additional benefit beyond exercise alone were concentrated in critically ill or acute-phase populations, or used lower-intensity or lower-frequency exercise modalities. Two studies were conducted in critically ill ICU cohorts during the ICU stay or the immediate post-ICU period despite high protein targets and resistance training (Wu et al. 2023 [15] and Wu et al. 2025 [39], both with HMB and 1.2–2.0 g/kg/day protein); one was conducted in geriatric medical inpatients with whey supplementation but only low-intensity resistance training [20]; one in stroke rehabilitation patients receiving vitamin D supplementation with standard rehabilitation [31]; and one in frail older adults receiving rehabilitation on only three non-consecutive days per week [27] (Supplementary Table S2). The clustering of null findings in critically ill cohorts and lower-intensity protocols is consistent with the hypothesis that high catabolic burden, illness severity, or insufficient exercise stimulus may attenuate the response to combined interventions.

### 3.4. Variability in Intervention Characteristics

Substantial heterogeneity was observed in both nutritional and exercise intervention characteristics across studies. Protein intake targets ranged widely, approximately from 0.66 to 2.0 g/kg/day, while exercise interventions varied considerably in modality, frequency, intensity, duration, and timing of initiation. Resistance training, mobilization, neuromuscular electrical stimulation, cycle ergometry, aerobic exercise, and multidisciplinary rehabilitation programs were commonly used. Exercise frequency ranged from several sessions per week to twice-daily interventions. Supplementary Figure S2 demonstrates the variability in intervention characteristics across studies. Although studies reporting favorable outcomes tended to include relatively higher protein intake and more intensive rehabilitation programs, considerable overlap remained among studies with positive and neutral findings.

Quantitatively, protein targets ranged from approximately 0.66 to 2.0 g/kg/day; exercise frequency from three sessions per week to twice-daily; session duration from 15 to 60 min; and timing of initiation from ICU day 1 to the rehabilitation phase (Supplementary Table S2). This degree of variation precluded the identification of a single optimal protocol.

Clinical patterns across these intervention characteristics were further explored by cross-tabulating reported outcome directions with intervention features. Four candidate moderating factors emerged from this descriptive cross-tabulation. First, population phase: favourable C versus D outcomes clustered in rehabilitation-phase populations (8 of 11 favourable studies), whereas null findings concentrated in critically ill ICU cohorts (2 of 5 unfavourable studies, plus the partial-benefit study [33] restricted to a less severely ill subgroup). Second, exercise modality: resistance training was present in 7 of 11 favourable studies versus more variable, lower-intensity, or non-resistance protocols in 4 of 5 unfavourable studies. Third, protein adequacy: explicit targets of ≥1.2 g/kg/day or ≥20 g/day whey/protein supplementation were reported in 5 of 11 favourable studies; notably, 3 of 5 unfavourable studies also achieved ≥ 1.2 g/kg/day [15,27,39] without observing an additional benefit beyond exercise, suggesting that protein adequacy alone is insufficient when illness severity is high or the exercise stimulus is limited. Fourth, protocol frequency and duration: five favourable studies used ≥ 5 sessions per week or comprehensive multidisciplinary programmes [22,23,30,38,41], whereas three of the five unfavourable studies used lower-frequency or lower-intensity protocols [20,27,31]. These patterns are descriptive observations derived from cross-tabulation of intervention characteristics with reported effect directions, and should not be interpreted as quantitative effect-modification estimates; they are presented as hypothesis-generating signposts for focused systematic reviews and adequately powered trials.

### 3.5. Evidence Gap Across Study Designs

**Figure 3:**
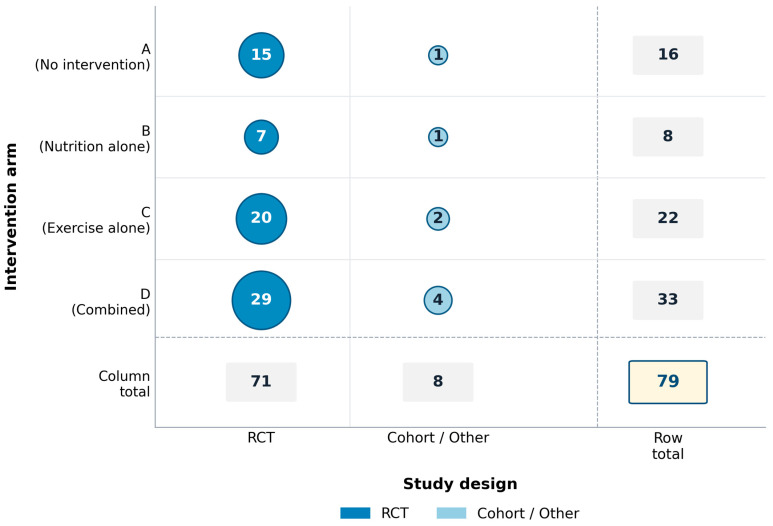
Evidence Gap Map of Nutrition and Exercise Interventions by Study Design. Distribution of intervention arms across the 33 included studies, stratified by study design. Each study contributes one count per intervention arm it includes (A: no intervention; B: nutrition alone; C: exercise alone; D: combined nutrition and exercise); studies with multiple arms are therefore represented in more than one cell, and the total number of arms (*n* = 79) exceeds the number of included studies (*n* = 33). Bubble area is approximately proportional to the number of arms; the exact count is shown within each bubble. Blue bubbles indicate study counts for randomized controlled trials and grey bubbles indicate cohort/other designs; bubble area is approximately proportional to the number of arms. Grey cells in the row and column margins show the row and column totals, and the yellow-highlighted cell shows the overall total number of intervention arms (*n* = 79).

Figure 3 demonstrates the distribution of available evidence according to intervention type and study design. Randomized controlled trials represented the majority of included studies, whereas cohort was relatively limited. The largest evidence cluster was observed among nutrition-only interventions, particularly within cohort studies. Exercise-only interventions were also relatively common across both randomized controlled trials and cohort studies. In contrast, studies evaluating combined nutrition and exercise interventions were limited across all study designs, especially rigorously designed randomized controlled trials directly com-paring combined and single-component strategies. These findings highlight a substantial evidence gap regarding the incremental and synergistic effects of combined nutrition and exercise interventions during hospitalization.

## 4. Discussion

This scoping review systematically mapped the available evidence on nutritional and exercise interventions during hospitalization in patients with frailty or sarcopenia, characterising the distribution of intervention configurations across the A–D framework and identifying gaps in the literature. Consistent with scoping review methodology, this review did not estimate intervention effectiveness; rather, it mapped the evidence landscape and signposted where future systematic reviews and meta-analyses are warranted. The main mapping observations were as follows: (1) the included studies were unevenly distributed across the four-group framework, with most evidence concentrated in configurations including an exercise component (Groups C and D) and relatively few studies isolating nutrition (Group B); (2) direct head-to-head comparisons between configurations were available for only a subset of contrasts (notably A versus D and C versus D), while B versus D comparisons were particularly scarce; and (3) populations, intervention dose, and outcome measures were highly heterogeneous, which both limits cross-study comparison and signals important areas for future standardisation.

A major finding of this review was that exercise interventions demonstrated relatively consistent associations with improvements in muscle strength, mobility, gait performance, and activities of daily living across various patient populations [16,18,24]. This finding is biologically plausible because exercise directly stimulates neuromuscular activation, muscle protein synthesis, and functional adaptation. In contrast, nutritional interventions alone often demonstrated inconsistent or limited effects [15,17,27]. Although adequate protein and energy intake are essential for preventing catabolism and supporting muscle maintenance, nutritional supplementation without sufficient anabolic stimulation may be insufficient to induce meaningful functional recovery during hospitalization. Previous studies have similarly suggested that nutritional support alone may have limited effects on muscle function unless combined with exercise-based rehabilitation [21,28].

Another important observation was the substantial heterogeneity in intervention characteristics across studies. Protein intake targets, energy delivery, exercise modality, rehabilitation frequency, intensity, duration, and timing of initiation varied considerably among studies. In particular, exercise interventions ranged from low-intensity mobilization to structured resistance training and multidisciplinary rehabilitation programs. This heterogeneity complicates interpretation of the literature and limits the ability to identify optimal intervention strategies. Supplementary Figure S2 clearly demonstrated that intervention dose and exercise prescription were not standardized across studies. Such variability likely contributed to the inconsistent findings regarding combined interventions and incremental effects. Previous rehabilitation literature has similarly highlighted the lack of standardization in exercise prescription and intervention reporting across clinical rehabilitation studies [19,26,38].

Importantly, relatively few studies were specifically designed to evaluate the incremental contribution of nutrition beyond exercise alone or vice versa. Although several studies reported favorable outcomes with combined interventions, many lacked appropriate comparator groups to determine whether the observed benefits were attributable to exercise, nutrition, or their interaction. The conceptual A–D framework used in this review enabled visualization of these comparison structures and highlighted the limited number of studies directly evaluating additive or synergistic effects [17,39]. This represents a major limitation in the current evidence base and may partly explain why the superiority of combined interventions over exercise alone remains uncertain.

This review has several important clinical implications. First, the findings support the importance of implementing structured exercise and mobilization programs during hospitalization whenever feasible [19,46]. Second, nutritional interventions should likely be integrated with rehabilitation strategies rather than provided in isolation [21,30]. Third, future intervention studies should adopt more standardized reporting of protein intake, energy delivery, rehabilitation intensity, and exercise dose to improve comparability across studies. Finally, future randomized controlled trials should directly compare combined and single-component interventions using factorial or incremental study designs to clarify the relative contribution of each component [15,16,17].

Mechanistically, exercise particularly resistance training enhances the anabolic sensitivity of skeletal muscle and counteracts the anabolic resistance characteristic of acute illness and aging, while adequate protein and amino acid intake provides the substrate necessary for muscle protein synthesis. These mechanisms are physiologically complementary, providing a rationale for why combined interventions may exceed the effect of either component alone, particularly when the anabolic stimulus from exercise is of sufficient intensity to render supplemental substrate effective.

As detailed in Section 3.3 and Section 3.4, cross-tabulation of intervention characteristics from Supplementary Table S2 with reported effect directions across the 17 C versus D contrasts identified four candidate moderating factors: population phase, exercise modality, protein adequacy, and protocol frequency or duration. Favourable effects clustered among rehabilitation-phase populations (8 of 11 favourable studies) using resistance-based exercise (7 of 11) with explicit protein targets of ≥1.2 g/kg/day or ≥20 g/day (5 of 11), whereas null findings concentrated in critically ill ICU cohorts or studies using lower-intensity or lower-frequency exercise. Notably, three of the five unfavourable studies achieved protein targets ≥ 1.2 g/kg/day [15,27,39] without observing additional benefit beyond exercise, suggesting that protein adequacy alone is insufficient when illness severity is high or the exercise stimulus is limited. These patterns are consistent with the hypothesis that high catabolic burden and illness severity attenuate responsiveness to combined interventions, and identify population phase, timing of initiation, exercise intensity, protein adequacy, and protocol duration as candidate effect modifiers warranting explicit evaluation in future trials and meta-analyses with sufficient statistical power.

Definitive separation of independent and interactive effects ultimately requires factorial (2 × 2) trial designs in which each component is randomized independently, such as that employed by Wu et al. [39]. Direct comparisons between combined and single-component interventions remain scarce, which constrains conclusions about the incremental value of combined interventions and, by extension, about the most resource-efficient strategy in hospital settings, where staffing, time, and nutritional resources are frequently constrained. Future trials should adopt standardized reporting frameworks such as the Consensus on Exercise Reporting Template (CERT) for exercise interventions and consistent reporting of achieved protein and energy delivery for nutritional interventions to enhance cross-study comparability and facilitate replication.

These mapping observations complement rather than duplicate recent systematic reviews and meta-analyses on closely related questions. Quantitative syntheses have estimated pooled effects for specific comparisons in defined populations (for example, combined nutrition–exercise versus exercise alone, or versus standard care, in selected older or sarcopenic populations) [47,48,49,50]. The present scoping review serves a different and complementary purpose: by mapping the full distribution of intervention configurations across diverse hospitalised populations and study designs, it identifies which contrasts and which clinical contexts have been studied densely enough to support meta-analysis, and which remain insufficiently studied. In particular, B versus D comparisons and direct comparisons in critically ill ICU cohorts emerge as priority targets for future randomised trials and subsequent meta-analytic synthesis. The descriptive patterns reported above therefore should not be interpreted as effectiveness estimates, but as signposts for where focused systematic reviews and adequately powered trials are most needed.

Several limitations should be acknowledged. First, as a scoping review, this study mapped the available evidence without performing quantitative synthesis or assessing intervention effectiveness; determination of the relative effectiveness of combined versus single-component interventions requires focused systematic reviews with meta-analysis in well-defined populations and comparison contrasts. Second, the included populations, settings, interventions, and outcomes were highly heterogeneous and addressed non-equivalent comparisons, which further precluded quantitative synthesis. Third, several included studies had small sample sizes and were exploratory or pilot in nature, limiting the certainty of the evidence. Fourth, intervention intensity and nutritional dose were inconsistently reported, limiting detailed comparison of exposure. Fifth, although PEDro scores indicated overall moderate-to-good methodological quality among the included RCTs (median 6/10; range 4–9), the methodological quality of some included studies was limited and risk of bias could not be fully excluded. Finally, publication bias and selective reporting may have influenced the available evidence.

## 5. Conclusions

Among the 33 included studies, most evaluated configurations including an exercise component, while studies isolating nutrition or directly comparing combined versus single-component interventions were limited. Populations, intervention dose, and comparison structures were highly heterogeneous, precluding quantitative synthesis. Consistent with scoping review methodology, this review mapped the evidence landscape rather than determining intervention effectiveness; B versus D comparisons and direct comparisons in critically ill ICU cohorts emerged as priority gaps. Adequately powered, factorial (2 × 2) randomized controlled trials with standardised reporting of intervention dose, together with focused systematic reviews and meta-analyses in well-defined populations and comparisons, are needed to advance the field.

## Figures and Tables

**Table 1:** Characteristics and outcomes of included studies.

Study	Country	Design	Population	Sample Size	Setting	Outcomes
Wu et al. (2023) [15]	China	RCT (4-arm)	Critically ill ICU patients	112	ICU	Physical performance; Clinical outcomes
Zak et al. (2009) [16]	Poland/Belgium	RCT (4-arm)	Frail older adults	91	ICU	ADL/Functional status; Physical performance; Muscle strength
Miller et al. (2006) [17]	Australia	RCT (4-arm)	Hospitalized older adults	100	Acute care ward	Nutritional outcomes; Muscle strength; Physical performance; QOL
Zong et al. (2023) [18]	China	RCT (3-arm)	Hospitalized patients with COPD	90	Rehabilitation ward	Muscle strength; Physical performance; QOL
Zhou et al. (2022) [19]	China	RCT (3-arm)	Critically ill ICU patients	150	ICU	Clinical outcomes; Muscle strength; ADL/Functional status
Gade et al. (2019) [20]	Denmark	RCT	Hospitalized older adults	141	Rehabilitation ward	Physical performance; ADL/Functional status; Muscle strength
Niccoli et al. (2017) [21]	Canada	RCT	Frail older adults	47	Rehabilitation ward	Muscle strength
Coiro et al. (2025) [22]	Switzerland	RCT	Hospitalized patients with COPD	52	Rehabilitation ward	Nutritional outcomes; Muscle strength
Kasahara et al. (2025) [23]	Japan	RCT	Hospitalized patients with cancer	42	Oncology ward	Muscle strength; Physical performance; QOL
Oyama et al. (2024) [24]	Japan	RCT	Hospitalized patients with COPD	38	Rehabilitation ward	Muscle strength; Physical performance
Opoda et al. (2024) [25]	Egypt	RCT	Critically ill ICU patients	70	ICU	Muscle mass; Clinical outcomes
Verceles et al. (2023) [26]	USA	RCT	Critically ill ICU patients	39	ICU	Muscle mass; Nutritional outcomes; Clinical outcomes
Strasser et al. (2023) [27]	Austria	RCT	Hospitalized older adults	40	Surgical ward	Muscle strength; Physical performance
Kamo et al. (2020) [28]	Japan	RCT	Hospitalized patients after liver transplantation	23	ICU	Muscle mass; Muscle strength
Nakamura et al. (2021) [29]	Japan	RCT	Critically ill ICU patients	117	ICU	Muscle mass; ADL/Functional status; QOL
Rondanelli et al. (2020) [30]	Italy	RCT	Hospitalized patients with sarcopenia	140	Rehabilitation ward	Physical performance; Muscle mass; ADL/Functional status
Momosaki et al. (2019) [31]	Japan	RCT	Hospitalized patients with stroke	100	Rehabilitation ward	ADL/Functional status; Muscle strength
Pedersen et al. (2019) [32]	Denmark	RCT	Hospitalized older adults	85	Acute care ward	Physical performance; Muscle strength; ADL/Functional status
Nakamura et al. (2020) [33]	Japan	RCT	Critically ill ICU patients	88	ICU	Muscle mass; ADL/Functional status
Strasser et al. (2020) [34]	Austria	RCT	Hospitalized patients with Hip fracture	40	Acute care ward	Immune biomarkers; Muscle strength
Veldsman et al. (2026) [35]	South Africa	RCT	Critically ill ICU patients	50	ICU	Muscle mass; Muscle strength; Physical performance
Walsh et al. (2015) [36]	UK	RCT	Critically ill ICU patients	240	Post-ICU ward	QOL; Muscle strength
Buhl et al. (2015) [37]	Denmark	RCT	Hospitalized older adults	29	Acute care ward	Muscle mass; ADL/Functional status
Yoshimura et al. (2016) [38]	Japan	RCT	Hospitalized older adults	39	Rehabilitation ward	Muscle strength; ADL/Functional status; Nutritional outcomes
Wu et al. (2025) [39]	China	RCT	Critically ill ICU patients	266	Post-ICU ward	Physical performance; Muscle mass; Muscle strength
Ng et al. (2024) [40]	Singapore	RCT	Critically ill ICU patients	21	ICU	Muscle strength; Muscle mass
Giovannini et al. (2024) [41]	Italy	RCT	Hospitalized patients with stroke	24	Rehabilitation ward	Physical performance; QOL
Nickels et al. (2024) [42]	Australia	RCT	Critically ill ICU patients	72	ICU	Muscle mass
Strasser et al. (2023) [27]	Austria	RCT	Frail older adults	20	Rehabilitation ward	Muscle strength; Nutritional outcomes; Muscle mass
Zhang et al. (2018) [43]	China	Cohort	Critically ill ICU patients	529	Acute care ward	Clinical outcomes
Zhou et al. (2024) [44]	China	Retrospective study	Hospitalized patients with cancer	68	Surgical ward	Nutritional outcomes; Clinical outcomes; Muscle function; QOL
Park et al. (2022) [45]	Korea	Comparative study	Hospitalized patients with stroke	54	Rehabilitation ward	Muscle mass; ADL/Functional status; Physical performance
Nakano et al. (2021) [46]	Japan	Historical study	Critically ill ICU patients	101	ICU	Muscle mass; ADL/Functional status

Abbreviations: RCT, Randomized controlled trial; ADL, activities of daily living; ICU, intensive care unit; QOL, quality of life. Population and setting terminology were standardized for presentation purposes. Outcome measures were grouped into broader domains, including muscle mass, muscle strength, physical performance, ADL/functional status, nutritional outcomes, clinical outcomes, immune biomarkers, and QOL.

## Data Availability

No new data were created or analyzed in this study. Data sharing is not applicable to this article.

## Supplementary Materials

The following supporting information can be downloaded at: https://www.mdpi.com/article/10.3390/nu18121994/s1. Table S1: Search strategy used for each database; Table S2: Characteristics of nutritional and exercise interventions; Table S3. Classification of interventions, comparison structure, and methodological quality (PEDro scores) of the 33 included studies; Figure S1: Outcome mapping across intervention categories; Figure S2: Variability in intervention characteristics across included studies.

## Abbreviations

The following abbreviations are used in this manuscript:

ADL	Activities of Daily Living
CENTRAL	Cochrane Central Register of Controlled Trials
COPD	Chronic Obstructive Pulmonary Disease
EMBASE	Excerpta Medica Database
ICU	Intensive Care Unit
OSF	Open Science Framework
PEDro	Physiotherapy Evidence Database
PRISMA-ScR	Preferred Reporting Items for Systematic Reviews and Meta-Analyses Extension for Scoping Reviews
QOL	Quality of Life
RCT	Randomized Controlled Trial
