# Supplementary material for: Nutrition and Exercise Interventions During Hospitalization in Frail or Sarcopenic Patients: A Scoping Review of Intervention Configurations and Evidence Gaps
Source: Nutrients. 2026 Jun 19;18(12):1994. doi: 10.3390/nu18121994 (PMC13306048; doi:10.3390/nu18121994)
Supplement: Supplementary file 1 [file nutrients-18-01994-s001.zip › nutrients-4347151-supplementary.pdf]

(Supplementary File 1)

"Nutrition and Exercise Interventions During Hospitalization in Frail or Sarcopenic Patients: A Scoping Review of In-tervention Configurations and Evidence Gaps"

**Supplementary File – Figures**

Supplemental Figure S1. Outcome Mapping Across Different Intervention Groups
Supplemental Figure S2. Variability in Intervention Characteristics Across Included Studies

**Supplementary File – Tables**

Supplementary Table S1: Search strategy used for each database
Supplementary Table S2: Characteristics of nutritional and exercise interventions
Supplementary Table S3. Classification of interventions, comparison structure, and methodological quality (PEDro scores) of the 33 included studies.

## Supplementary Table S1. Search strategy used for each database

The search strategy was developed using a combination of controlled vocabulary terms and free-text keywords related to hospitalization, frailty or sarcopenia, nutrition, and exercise or rehabilitation. The final searches were conducted in April 2026 across the included databases (PubMed/MEDLINE: 11 April 2026; EMBASE: 10 April 2026; PEDro: 15 April 2026; CENTRAL: 13 April 2026). The term outpatient was retained in some database searches to maximize sensitivity; however, studies conducted exclusively in outpatient or community settings were excluded during screening.

| Database | Search Strategy |
|---|---|
| **PubMed/MEDLINE** | #1 **Setting / hospitalization**: ("Hospitalization"[mh] OR hospitalization[tiab] OR hospitalized[tiab] OR "Inpatients"[mh] OR inpatient*[tiab] OR outpatient*[tiab] OR "Acute Disease"[mh] OR "acute illness"[tiab] OR "Critical Illness"[mh] OR "critical illness"[tiab] OR "critically ill"[tiab] OR ICU[tiab] OR "intensive care"[tiab] OR "bed rest"[tiab] OR bedridden[tiab])<br>#2 **Frailty / sarcopenia**: ("Frailty"[mh] OR frail*[tiab] OR "Sarcopenia"[mh] OR sarcopenia[tiab] OR "muscle loss"[tiab] OR "muscle wasting"[tiab] OR "low muscle mass"[tiab] OR "muscle mass"[tiab] OR "muscle weakness"[tiab] OR "lean body mass"[tiab] OR "ICU-acquired weakness"[tiab] OR "acquired weakness"[tiab] OR ICUAW[tiab] OR "physical recovery"[tiab])<br>#3 **Nutrition**: ("Nutrition Therapy"[mh] OR "Nutritional Support"[mh] OR "Dietary Supplements"[mh] OR "Enteral Nutrition"[mh] OR "Parenteral Nutrition"[mh] OR nutrition*[tiab] OR dietary[tiab] OR protein*[tiab] OR "nutritional support"[tiab] OR "energy intake"[tiab] OR "oral nutritional supplement*"[tiab] OR "essential amino acid*"[tiab] OR amino acid*[tiab] OR "enteral nutrition"[tiab] OR "parenteral nutrition"[tiab] OR "high protein"[tiab]) |

| Database | Search Strategy |
|---|---|
| | #4 **Exercise / rehabilitation**: ("Rehabilitation"[mh] OR "Exercise"[mh] OR "Exercise Therapy"[mh] OR "Physical Therapy Modalities"[mh] OR rehabilitation[tiab] OR exercise*[tiab] OR "physical therapy"[tiab] OR physiotherapy[tiab] OR mobilization[tiab] OR mobilisation[tiab] OR "early mobilization"[tiab] OR ambulation[tiab] OR walking[tiab])<br>#5 **Final search**: #1 AND #2 AND #3 AND #4 |
| **EMBASE** | #1 **Setting / hospitalization**: ('hospitalization'/exp OR hospitalization:ti,ab OR hospitalized:ti,ab OR 'inpatient'/exp OR 'acute disease'/exp OR 'acute illness':ti,ab OR 'critical illness'/exp OR 'critical illness':ti,ab OR 'critically ill':ti,ab OR ICU:ti,ab OR 'intensive care':ti,ab OR 'bed rest':ti,ab OR bedridden:ti,ab)<br>#2 **Frailty / sarcopenia**: ('frailty'/exp OR frail*:ti,ab OR 'sarcopenia'/exp OR sarcopenia:ti,ab OR 'muscle loss':ti,ab OR 'muscle wasting':ti,ab OR 'low muscle mass':ti,ab OR 'muscle weakness':ti,ab OR 'lean body mass':ti,ab OR 'intensive care unit acquired weakness'/exp OR 'ICU-acquired weakness':ti,ab OR 'acquired weakness':ti,ab OR ICUAW:ti,ab OR 'physical recovery':ti,ab)<br>#3 **Nutrition**: ('nutrition therapy'/exp OR 'nutritional support'/exp OR 'dietary supplement'/exp OR 'enteral nutrition'/exp OR 'parenteral nutrition'/exp OR nutrition*:ti,ab OR dietary:ti,ab OR 'nutritional support':ti,ab OR 'energy intake':ti,ab OR 'oral nutritional supplement':ti,ab OR 'oral nutritional supplements':ti,ab OR 'essential amino acid':ti,ab OR 'essential amino acids':ti,ab OR 'amino acid':ti,ab OR 'amino acids':ti,ab OR 'enteral nutrition':ti,ab OR 'parenteral nutrition':ti,ab OR 'high protein':ti,ab)<br>#4 **Exercise / rehabilitation**: ('rehabilitation'/exp OR 'exercise'/exp OR 'exercise therapy'/exp OR 'physical therapy'/exp OR rehabilitation:ti,ab OR exercise*:ti,ab OR 'physical therapy':ti,ab OR physiotherapy:ti,ab OR mobilization:ti,ab OR mobilisation:ti,ab OR 'early mobilization':ti,ab OR ambulation:ti,ab OR walking:ti,ab)<br>#5 **Final search**: #1 AND #2 AND #3 AND #4 |
| **PEDro** | The PEDro database was searched using combinations of keywords related to frailty, sarcopenia, nutrition, and exercise. Due to the limitations of the PEDro search interface, searches were conducted using simplified keyword combinations, and results were screened manually for eligibility.<br>#1 **Title and Abstract**: frailty rehabilitation nutrition<br>#2 **Title and Abstract**: sarcopenia exercise nutrition<br>#3 **Title and Abstract**: ICU rehabilitation nutrition<br>#4 **Filter**: Clinical trial<br>#5 **Subdiscipline**: Geriatrics |

| Database | Search Strategy |
| --- | --- |
| **CENTRAL (Cochrane Central Register of Controlled Trials)** | #**1 Setting / hospitalization**: ([mh "Hospitalization"] OR hospitalization:ti,ab,kw OR hospitalized:ti,ab,kw OR [mh "Inpatients"] OR inpatient*:ti,ab,kw OR outpatient*:ti,ab,kw OR [mh "Acute Disease"] OR "acute illness":ti,ab,kw OR [mh "Critical Illness"] OR "critical illness":ti,ab,kw OR "critically ill":ti,ab,kw OR ICU:ti,ab,kw OR "intensive care":ti,ab,kw OR "bed rest":ti,ab,kw OR bedridden:ti,ab,kw)<br>#**2 Frailty / sarcopenia:** ([mh "Frailty"] OR frail*:ti,ab,kw OR [mh "Sarcopenia"] OR sarcopenia:ti,ab,kw OR "muscle loss":ti,ab,kw OR "muscle wasting":ti,ab,kw OR "low muscle mass":ti,ab,kw OR "muscle mass":ti,ab,kw OR "muscle weakness":ti,ab,kw OR "lean body mass":ti,ab,kw OR "ICU-acquired weakness":ti,ab,kw OR "acquired weakness":ti,ab,kw OR ICUAW:ti,ab,kw OR "physical recovery":ti,ab,kw)<br>#**3 Nutrition:** ([mh "Nutrition Therapy"] OR [mh "Nutritional Support"] OR [mh "Dietary Supplements"] OR [mh "Enteral Nutrition"] OR [mh "Parenteral Nutrition"] OR nutrition*:ti,ab,kw OR dietary:ti,ab,kw OR protein*:ti,ab,kw OR "nutritional support":ti,ab,kw OR "energy intake":ti,ab,kw OR (oral NEXT nutritional NEXT supplement*):ti,ab,kw OR (essential NEXT amino NEXT acid*):ti,ab,kw OR (amino NEXT acid*):ti,ab,kw OR "enteral nutrition":ti,ab,kw OR "parenteral nutrition":ti,ab,kw OR "high protein":ti,ab,kw)<br>#**4 Exercise / rehabilitation:** ([mh "Rehabilitation"] OR [mh "Exercise"] OR [mh "Exercise Therapy"] OR [mh "Physical Therapy Modalities"] OR rehabilitation:ti,ab,kw OR exercise*:ti,ab,kw OR "physical therapy":ti,ab,kw OR physiotherapy:ti,ab,kw OR mobilization:ti,ab,kw OR mobilisation:ti,ab,kw OR "early mobilization":ti,ab,kw OR ambulation:ti,ab,kw OR walking:ti,ab,kw)<br>#**5 Final search:** #1 AND #2 AND #3 AND #4 |

**Supplementary Table S2. Characteristics of nutritional and exercise interventions**

| Study | Nutrition Type | Protein | Energy | Exercise Type | Frequency | Intensity | Duration | Initiation |
|---|---|---|---|---|---|---|---|---|
| Wu et al. (2023) [15] | HMB (3 g/day) | 1.2–2.0 g/kg/day | 20–30 kcal/kg/day | RT | 5 times/week | Moderate | 20–30 min | ICU–ward |
| Zak et al. (2009) [16] | Multi-nutrient supplementation | 16% of 300 kcal from protein | 300 kcal/day supplementation | RT + FOE / SE | 5 times/week | Moderate–high | 45 min | During intervention |
| Miller et al. (2006) [17] | Oral nutritional supplement | 16% protein | Energy-dense (6.3 kJ/mL) | RT | 3 times/week | Moderate | 20–30 min | Day 7 post-fracture |
| Zong et al. (2023) [18] | Whey protein supplementation | 10 g whey protein per packet | Not clearly reported | Low-intensity aerobic + RT | 4 times/week | Low–moderate | 20 min | Rehab phase |
| Zhou et al. (2022) [19] | Early nutrition | 1.3 g/kg | 20–25 kcal/kg | Early mobilization | Twice daily | Progressive | 20–30 min/session | Day 1-2 |
| Gade et al. (2019) [20] | Whey protein supplement | 27.5 g/day | ~2000 kJ/day | Low-intensity RT | Daily (hospital) | Low | NR | Admission |
| Niccoli et al. (2017) [21] | Whey protein | 24 g/day | NR | Geriatric rehabilitation | Daily | Low–moderate | NR | Admission |
| Coiro et al. (2025) [22] | Individual nutritional therapy | Target 1.2–1.5 g/kg/day | TEE-based | Endurance + RT | 5 times/week + 2–3 times/week (RT) | Moderate | 15–30 min | Admission |
| Kasahara et al. (2025) [23] | ONS (included BCAA) | 20 g/day protein | 200 kcal/day | RT + aerobic exercise | 6 times/week | Moderate (Borg 13) | 20 min | During chemotherapy |
| Oyama et al. (2024) [24] | ONS (included BCAA) | ~6.5 g/pack | TEE-based | Early rehabilitation | NR | Moderate | 15 min | Day 3 |
| Opoda et al. (2024) [25] | High protein vs standard | 1.2 g/kg/day vs 2.0 g/kg/day | 20–25 kcal/kg | Early exercise | Daily | Progressive | 20 min | Day 2 |
| Verceles et al. (2023) [26] | High protein supplementation | 1.3 ± 0.4 g/kg/day | 20.1 ± 7.5 kcal/kg/day | NMES + PT + mobility | Daily | Moderate | 30 min/session | ICU stay |
| Strasser et al. (2020) [27] | Protein-enriched diet + drink | ~1.2 g/kg/day | Supplement provided 300 kcal/day | RT | 3 times/week | Moderate | 30 min | During hospitalization |
| Kamo et al. (2020) [28] | HMB + amino acids | 1.2–1.5 g/kg/day | 25–30 kcal/kg | Rehabilitation + early mobilization | Daily | Progressive | NR | POD 1 |
| Nakamura et al. (2021) [29] | High vs medium protein | 1.8 g/kg/day vs 0.9 g/kg/day | 20 kcal/kg | Rehabilitation + EMS | Daily | Moderate | 20 min | Day 2 |
| Rondanelli et al. (2020) [30] | Whey protein + leucine + vitamin D | 20 g/serving ×2/day | 150 kcal/serving | RT-based rehabilitation | 5 times/week | Moderate (Borg 12–14) | 20–30 min | Hospital admission |
| Momosaki et al. (2019) [31] | Vitamin D only | NR | NR | Standard rehab | Daily | NR | NR | Hospital admission |
| Pedersen et al. (2019) [32] | Protein supplement post-exercise | 18 g/session | 300 kcal | Strength training | 3 times/week | Moderate–high | ~20 min | Admission |
| Nakamura et al. (2019) [33] | HMB + arginine + glutamine supplementation | Total protein about 1.0 g/kg/day | 20–30 kcal/kg | EMS + rehabilitation | Daily | Moderate | 20 min | Day 2 |

| Study | Nutrition Type | Protein | Energy | Exercise Type | Frequency | Intensity | Duration | Initiation |
|---|---|---|---|---|---|---|---|---|
| Strasser et al. (2020) [34] | High protein diet | 1.2 g/kg | ~1400 kcal | Resistance training | 3 times/week | Moderate | 30 min | During stay |
| Veldsman et al. (2026) [35] | Intravenous amino acid supplementation | +0.37 g/kg/day(total ~1.57 g/kg) | 20–25 kcal/kg/day | Cycle ergometry | Daily | Moderate | 45 min | Day 3–4 ICU |
| Walsh et al. (2015) [36] | Increased dietetic assessment | NR | NR | Increased rehabilitation dose | 2–3-fold higher frequency | 2–3-fold higher frequency | Not clearly reported | Post-ICU |
| Buhl et al. (2015) [37] | High-protein diet + ONS | 1.7 g/kg/day | Weight maintenance | RT | 3 times/week | Moderate | Not clearly reported | During hospitalization |
| Yoshimura et al. (2016) [38] | Nutritional supplementation | NR | Adequate intake ensured | Resistance training | 3 times/week | Moderate | Up to 3 h/day | Inpatient rehabilitation |
| Wu et al. (2025) [39] | HMB | 1.2–2.0 g/kg/day | 20–30 kcal/kg/day | RT | 5 times/week | Moderate (Borg 11–13) | 20–30 min | ICU early |
| Ng et al. (2024) [40] | Indirect calorimetry feeding | Median 1.6 g/kg/day | ~23 kcal/kg/day | Cycle ergometry | Daily | Moderate | 60 min | ICU early |
| Giovannini et al. (2024) [41] | Vitamins + minerals | NR | NR | Intensive multidisciplinary rehabilitation | 6 times/week | Moderate | 2h/day | Rehabilitation admission |
| Nickels et al. (2024) [42] | Usual ICU nutrition | Mean 0.66 g/kg/day | NR | In-bed cycling | 6 times/week | Moderate | 30 min | ICU early |
| Strasser et al. (2023) [43] | Protein-enriched diet | 1.2–1.5 g/kg/day | 150 kcal/serving × 2/day | Standard rehabilitation | 3 non-consecutive days/week | Low–moderate | 30 min | Admission |
| Zhang et al. (2018) [44] | Nutrition support | 0.25 g nitrogen/kg/day | 50 kJ/kg/day non-protein calories | Early rehab | Daily | Progressive | NR | POD 1 |
| Zhou et al. (2024) [45] | Personalized enteral nutrition | NR | NR | Personalized exercise | Daily | Moderate | 20–30 min | 7 days before surgery |
| Park et al. (2022) [46] | BCAA supplementation | ~12 g/day | ~30 kcal/kg/day | Intensive rehabilitation | Daily | Moderate | NR | Early rehabilitation |
| Nakano et al. (2021) [47] | High-protein protocol | Target 1.8 g/kg/day | 20–30 kcal/kg/day | Mobilization + NMES | Daily | Progressive | 20 min | Day 1 ICU |

Abbreviations: BCAA, branched-chain amino acid; EMS, electrical muscle stimulation; FOE, functionally oriented exercise; HMB, β-hydroxy-β-methyl butyrate; ICU, intensive care unit; NMES, neuromuscular electrical stimulation; NR, not reported; ONS, oral nutritional supplement; POD, postoperative day; PT, physical therapy; QOL, quality of life; RT, resistance training; SE, standard exercise; TEE, total energy expenditure.

**Supplementary Table S3. Classification of interventions, comparison structure, and methodological quality (PEDro scores) of the 33 included studies.**

| Study | A (None) | B (Nutr.) | C (Exer.) | D (Comb.) | Main findings / Outcome direction | PEDro score |
|---|---|---|---|---|---|---|
| Wu et al. (2023) [15] | ✓ | ✓ | ✓ | ✓ | B vs A: No / C vs A: Yes / D vs A: Yes / D vs C: No significant difference | **8/10** |
| Zak et al. (2009) [16] | ✓ | ✓ | ✓ | ✓ | B vs A: Limited / C vs A: Yes / D vs A: Yes / D vs C: Additional benefit for mobility/6MWT | 5/10 |
| Miller et al. (2006) [17] | ✓ | ✓ | ✓ | ✓ | B vs A: No / C vs A: No / D vs A: Partial / D vs C: Yes (less weight loss) | **8/10** |
| Zong et al. (2023) [18] | ✓ | – | ✓ | ✓ | C vs A: Yes / D vs A: Yes / D vs C: Additional benefit for dyspnea, anxiety, and weight | 4/10 |
| Zhou et al. (2022) [19] | ✓ | – | ✓ | ✓ | C vs A: Yes / D vs A: Yes / D vs C: No significant difference (muscle strength slightly better in D) | **7/10** |
| Gade et al. (2019) [20] | – | – | ✓ | ✓ | D vs C: No additional benefit of protein supplementation | **9/10** |
| Niccoli et al. (2017) [21] | – | – | ✓ | ✓ | D vs C: Yes (greater improvement in muscle strength and knee extensor force) | **7/10** |
| Coiro et al. (2025) [22] | – | – | ✓ | ✓ | D vs C: Yes (energy/protein intake and handgrip strength improved) | **7/10** |
| Kasahara et al. (2025) [23] | – | – | ✓ | ✓ | D vs C: Yes (improved muscle strength) | 5/10 |
| Oyama et al. (2024) [24] | – | ✓ | ✓ | ✓ | D vs C: Yes (quadriceps strength, gait speed, and muscle mass maintenance improved) | 5/10 |
| Opoda et al. (2024) [25] | – | – | – | ✓ | Higher-protein D group: Yes (better RF muscle CSA/thickness, shorter ICU stay, lower infection) | 5/10 |
| Verceles et al. (2023) [26] | ✓ | – | – | ✓ | D vs A: Yes (less muscle loss, improved nitrogen balance, less delirium) | 4/10 |
| Strasser et al. (2020) [27] | ✓ | – | – | ✓ | D vs A: Yes (muscle strength improved) | 6/10 |
| Kamo et al. (2020) [28] | – | – | ✓ | ✓ | D vs C: Yes (muscle strength, SMI, shorter hospital stay) | 6/10 |
| Nakamura et al. (2021) [29] | – | ✓ | ✓ | ✓ | D vs B: Yes (less femoral muscle loss, lower PIICS incidence) | 6/10 |
| Rondanelli et al. (2020) [30] | – | – | ✓ | ✓ | D vs C: Yes (gait speed, muscle mass, ADL, shorter rehabilitation stay) | **9/10** |
| Momosaki et al. (2019) [31] | – | – | ✓ | ✓ | D vs C: No (no improvement in Barthel Index, grip strength, calf circumference) | **9/10** |

| Study | A (None) | B (Nutr.) | C (Exer.) | D (Comb.) | Main findings / Outcome direction | PEDro score |
|---|---|---|---|---|---|---|
| Pedersen et al. (2019) [32] | ✓ | – | – | ✓ | D vs A: Partial (increased daily steps only; no significant mobility improvement) | **7/10** |
| Nakamura et al. (2019) [33] | – | – | ✓ | ✓ | D vs C: Partial (reduced muscle loss only in SOFA <10 subgroup) | 6/10 |
| Strasser et al. (2020) [34] | ✓ | – | – | ✓ | D vs A: Partial (improvement in muscle strength, no immune biomarker improvement) | 6/10 |
| Veldsman et al. (2026) [35] | ✓ | – | – | ✓ | D vs A: No | **7/10** |
| Walsh et al. (2015) [36] | ✓ | – | – | ✓ | D vs A: No | **7/10** |
| Buhl et al. (2015) [37] | ✓ | – | – | ✓ | D vs A: No | 5/10 |
| Yoshimura et al. (2016) [38] | – | – | ✓ | ✓ | D vs C: Yes (CC, AC, BI, Alb improved) | 5/10 |
| Wu et al. (2025) [39] | ✓ | ✓ | ✓ | ✓ | C improved physical function/mortality; D showed no additional synergistic benefit | **8/10** |
| Ng et al. (2024) [40] | ✓ | – | – | ✓ | D vs A: Trend only (no significant difference) | 6/10 |
| Giovannini et al. (2024) [41] | – | – | ✓ | ✓ | D vs C: Yes (fatigue, balance, cognition, body composition) | 6/10 |
| Nickels et al. (2024) [42] | ✓ | ✓ | ✓ | ✓ | No (muscle loss) | 4/10 |
| Strasser et al. (2023) [43] | – | – | ✓ | ✓ | D vs C: No | 5/10 |
| Zhang et al. (2018) [44] | – | ✓ | – | ✓ | D vs B: Yes (hospital stay, inflammatory markers, GI recovery, adverse events, survival) | *N/A* [1] |
| Zhou et al. (2024) [45] | – | – | ✓ | ✓ | Yes (nutrition, GI recovery, muscle function, QOL) | *N/A* [1] |
| Park et al. (2022) [46] | – | – | ✓ | ✓ | Yes (SMI, ADL, balance, gait, swallowing) | *N/A* [1] |
| Nakano et al. (2021) [47] | ✓ | – | – | ✓ | Yes (reduced femoral muscle loss, earlier mobilization) | *N/A* [1] |

[1] N/A: PEDro scoring is not applicable to non-randomized study designs (cohort, retrospective, comparative, and historical studies; refs [44]–[47]).

PEDro scores were obtained from the Physiotherapy Evidence Database (https://pedro.org.au); studies not indexed in PEDro were independently scored by two reviewers, with disagreements resolved by consensus. Scores range from 0 to 10, with higher scores indicating better methodological quality.

Summary of PEDro scores (n = 29 RCTs): range 4–9; median 6; mean 6.2. Distribution: 9 (n = 3); 8 (n = 3); 7 (n = 6); 6 (n = 6); 5 (n = 8); 4 (n = 3).

Abbreviations: 6MWT, 6-minute walk test; AC, arm circumference; ADL, activities of daily living; Alb, albumin; BI, Barthel Index; CC, calf circumference; CSA, cross-sectional area; GI, gastrointestinal; HMB, β-hydroxy-β-methyl butyrate; ICU, intensive care unit; PIICS, persistent inflammation, immunosuppression, and catabolism syndrome; QOL, quality of life; RF, rectus femoris; SMI, skeletal muscle index; SOFA, sequential organ failure assessment.

| Outcome | **A**<br>Nutrition absent<br>Exercise absent<br>(Control) | **B**<br>Nutrition present<br>Exercise absent<br>(Nutrition-only) | **C**<br>Nutrition absent<br>Exercise present<br>(Exercise-only) | **D**<br>Nutrition present<br>Exercise present<br>(Combined intervention) |
|---|---|---|---|---|
| **Muscle mass** | − | ± | ± | + |
| **Strength** | − | ± | + | ++ |
| **ADL**<br>(Activities of Daily Living) | − | ± | + | ++ |
| **LOS**<br>(Length of Stay) | − | ± | ± | + |

− Worse (-)  ± No change (±)  + Moderate improvement (+)  ++ Marked improvement (++)

**Supplemental Figure S1. Outcome Mapping Across Different Intervention Groups**

Outcome mapping across intervention categories. The figure summarizes the overall direction of reported outcomes according to intervention type. A: no intervention (control), B: nutrition-only intervention, C: exercise-only intervention, and D: combined nutrition and exercise intervention. Symbols indicate the relative direction of outcomes compared with the control group: −, worse outcome; ±, no clear difference; +, improvement; ++, marked improvement. Overall, exercise-containing interventions (C and D) were more consistently associated with improvements in muscle strength, physical function, mobility, and activities of daily living than nutrition-only interventions (B). Combined interventions (D) frequently demonstrated favorable outcomes; however, incremental benefits beyond exercise alone remained inconsistent across studies. Outcome classification was based on the original authors' reported conclusions for the primary outcome of each comparison. Positive findings were classified as + or ++, neutral findings as ±, and negative findings as −. Effect sizes and confidence intervals were not re-analyzed.

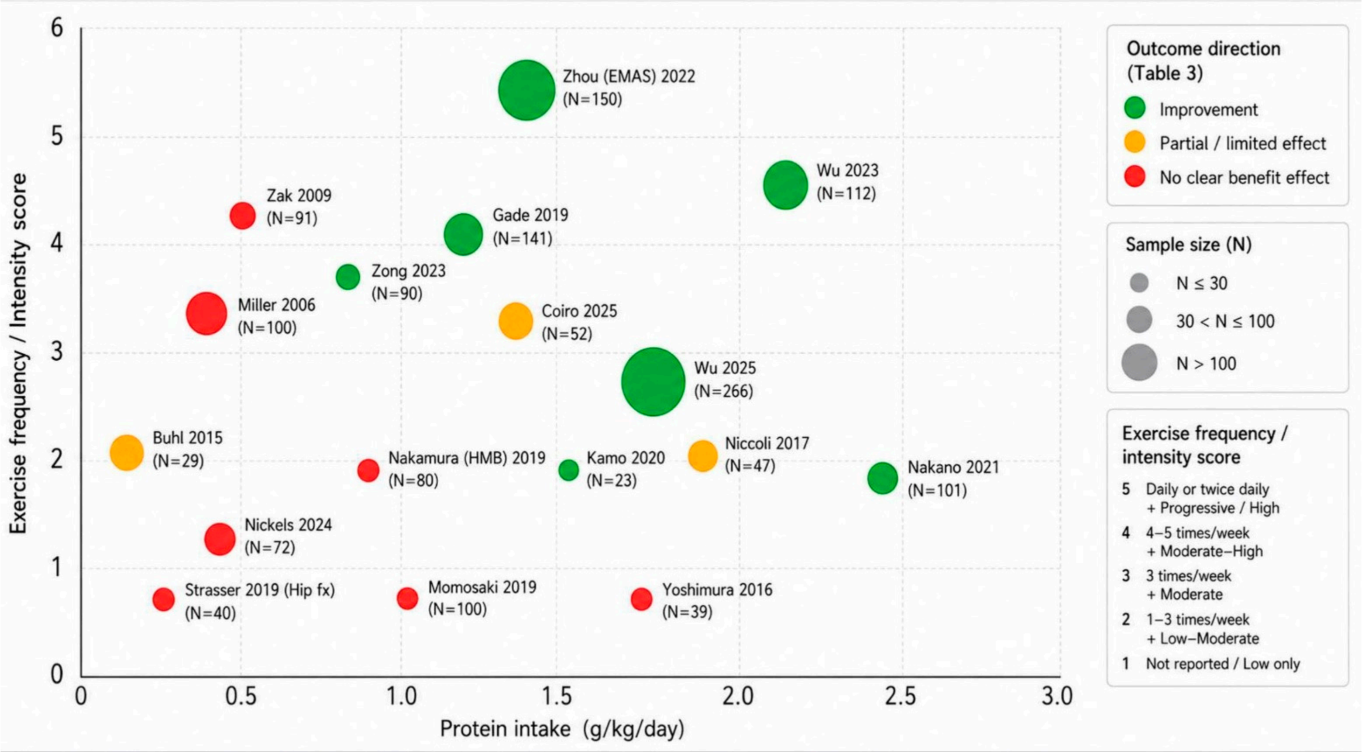


**Supplemental Figure S2. Variability in Intervention Characteristics Across Included Studies**
Bubble plot demonstrating heterogeneity in nutritional and exercise interventions across included studies. The x-axis represents protein intake (g/kg/day), and the y-axis represents exercise frequency/intensity score derived from intervention characteristics reported in Table S2. Bubble size corresponds to study sample size. Bubble colors indicate overall outcome direction based on Table 3 classifications: Green: improvement. Yellow: partial or limited benefit Red: no clear benefit. Exercise frequency/intensity score:1 = not reported or low only2 = 1–3 sessions/week with low–moderate intensity3 = 3 sessions/week with moderate intensi-ty4 = 4–5 sessions/week with moderate–high intensity5 = daily or twice-daily interventions with progressive/high intensity.
